# Inadequacy of Immune Health Nutrients: Intakes in US Adults, the 2005–2016 NHANES

**DOI:** 10.3390/nu12061735

**Published:** 2020-06-10

**Authors:** Carroll A. Reider, Ray-Yuan Chung, Prasad P. Devarshi, Ryan W. Grant, Susan Hazels Mitmesser

**Affiliations:** Science & Technology, Pharmavite LLC, West Hills, CA 91304, USA; raychung@umich.edu (R.-Y.C.); pdevarshi@pharmavite.net (P.P.D.); rgrant@pharmavite.net (R.W.G.); smitmesser@pharmavite.net (S.H.M.)

**Keywords:** micronutrient, immune, NHANES, vitamin A, vitamin C vitamin D, vitamin E, zinc, dietary supplements, nutritional adequacy, nutrient shortfalls, nutrient deficiencies

## Abstract

A well-functioning immune system is essential for human health and well-being. Micronutrients such as vitamins A, C, D, E, and zinc have several functions throughout the immune system, yet inadequate nutrient intakes are pervasive in the US population. A large body of research shows that nutrient inadequacies can impair immune function and weaken the immune response. Here, we present a new analysis of micronutrient usual intake estimates based on nationally representative data in 26,282 adults (>19 years) from the 2005–2016 National Health and Nutrition Examination Surveys (NHANES). Overall, the prevalence of inadequacy (% of population below estimated average requirement [EAR]) in four out of five key immune nutrients is substantial. Specifically, 45% of the U.S. population had a prevalence of inadequacy for vitamin A, 46% for vitamin C, 95% for vitamin D, 84% for vitamin E, and 15% for zinc. Dietary supplements can help address nutrient inadequacy for these immune-support nutrients, demonstrated by a lower prevalence of individuals below the EAR. Given the long-term presence and widening of nutrient gaps in the U.S.—specifically in critical nutrients that support immune health—public health measures should adopt guidelines to ensure an adequate intake of these micronutrients. Future research is needed to better understand the interactions and complexities of multiple nutrient shortfalls on immune health and assess and identify optimal levels of intake in at-risk populations.

## 1. Introduction

The immune system is made up of a large and complex network of cells, tissues organs, and systems throughout the body. It has two major components: innate and adaptive immunity. Innate immunity serves as the first line of defense, consisting of physical barriers that help block the entry of pathogens (eyes, skin, mucus membranes, and the epithelium of the gut), immune cells, and the complement system. The cells of the innate immune system, including phagocytes, neutrophils, dendritic cells and natural killer cells (NK), play critical roles in the body’s defenses by neutralizing pathogens and facilitating the adaptive immune response. The adaptive immune system, comprised of T and B cells, mounts a response to specific pathogens and develops immunological memory for future immune challenge. In the innate system, antigen-presenting cells facilitate the development of specific adaptive responses [1]. Micronutrients are critical for every stage of the immune response. For instance, vitamin D is known to trigger the production of antimicrobial peptides, and folate, vitamin B6 and B12 are all required for white blood cell production. Additionally, vitamins A, C, D, E, and zinc, iron, and selenium are all involved in the innate and/or adaptive immunity immune response. Intake of these vitamins and minerals from the diet supports the body’s initial and adaptive responses required for defense against pathogens [2,3]. Unfortunately, there is a high prevalence of inadequate intake of many of these nutrients in the U.S. population. The 2015–2020 U.S. Dietary Guidelines (DGAs) has identified underconsumed intake of vitamins A, C, D, and E across all populations, and iron for women of childbearing age [4]. Both the 2010 and 2015–2020 DGAs identified the inadequate intake of vitamin D as a public health issue [4,5]. Previous NHANES reports showed that for vitamins A, C, D, and E, a high percentage of the population fell below the estimated average requirement (EAR), a nutrient intake value that is estimated to meet the requirement of half the healthy individuals to avoid symptoms of a clinical or subclinical deficiency [6,7,8]. Zinc has a large prevalence of inadequacy in older adults [9]. Another lifestyle factor that can affect the immune system is inadequate sleep. Approximately 1/3 of US adults do not meet the recommended number of sleep hours. A recent report showed a high prevalence of vitamins A, C, D, E, and zinc inadequacies are significantly associated with inadequate sleep [10], which suggested nutrients play interdisciplinary roles in multiple systems that support immune health. Micronutrient inadequacy of these critical immune nutrients is also an important global health concern. The World Health Organization has classified the deficiency of vitamin A as a public health issue, especially in children and pregnant women, in over 50% of all countries [11]. Vitamin C inadequacy is common in many countries, specifically with at-risk populations [12], and almost 1 billion people around the world have low vitamin D levels, regardless of ethnicity or age [13]. Only 1/5 of the global population are at the optimal vitamin E status levels [14]. In developing countries, zinc deficiency is a health concern [15]. A large body of research shows micronutrient insufficiencies/deficiencies and inadequate intake can impair immune function and weaken immune response, which may increase the risk of infections and other immune-associated diseases and conditions [2,3,12,16,17,18,19,20,21,22,23,24,25,26,27,28,29,30,31,32,33,34,35,36,37,38,39,40,41].

The objectives of this paper are to present an analysis of a large cross-sectional U.S. population database on dietary intake, identify the current prevalence of nutrient inadequacies of key micronutrients critical for immune function, review the function of these shortfall nutrients important for the immune system, and discuss strategies for filling dietary nutrient gaps. Although many essential nutrients are important for immune health, we have chosen to focus this manuscript on vitamins A, C, D, and E due to the substantial nutrient inadequacies we found in the U.S. population, as well as zinc, which was the highest shortfall of the immune-related minerals.

## 2. Methods

The National Health and Nutrition Examination Survey (NHANES) is a bi-yearly cross-sectional study of the US population conducted by Centers for Disease Prevention and Control (CDC). Data from the 2005–2016 (2005–2006, 2007–2008, 2009–2010, 2011–2012, 2013–2014, and 2015–2016) NHANES was previously analyzed [10]. Details regarding the study participants, demographics, methods, statistical analyses are described by Ikonte et al. [10]. Briefly, the usual intake, % of population below EAR and % of population above the UL were analyzed using two reliable 24 h dietary recall interviews in 26,282 adults aged 19–99 years.

## 3. Prevalence of Immune-Related Micronutrient Inadequacies

Dietary reference intakes (DRIs) is a term used for a group of nutrient reference values set by the National Academy of Medicine. The set of four values are the estimated average requirement (EAR), recommended dietary allowance (RDA), adequate intake (AI), and the tolerable upper intake level (UL) [42]. The EAR is a nutrient intake value that is estimated to meet the requirement of 50% of healthy individuals in a group, while the RDA is set to provide the nutrient requirements of 97–98% of healthy individuals in a group [42]. EARs are determined using adequacy values that prevent deficiency signs or symptoms or rely on biomarkers of a nutrient’s function or nutrient body stores [8]. The prevalence of nutrient inadequacy in population data (NHANES) is determined by assessing the percent of the population that has a nutrient usual intake below the EAR [42].

The high prevalence of micronutrient inadequacy is a public health concern [4]. Per the Dietary Guidelines for Americans and the intake estimates based on the NHANES data, vitamins A, C, D, and E are the largest essential micronutrient inadequacies across all population groups [4,6,7]. Zinc has a large prevalence of inadequacy in older adults [9]. Older adults are at greater risk of impaired immune function with shifts in microbiome, increased medication intake, involution of the thymus and lower T cell production and multiple underlying health conditions—all of which increase risk of infection [43].

### 3.1. Prevalence of Inadequate Intake from Food Only, the 2005–2016 NHANES 

Our study shows that a significant number of Americans did not meet recommendations for micronutrients essential for immunocompetence. Specifically, in all adults (>19 years), 45% of the U.S. population had a prevalence of inadequacy (% of population below EAR) for vitamin A, 46% for vitamin C, 95% for vitamin D, 84% for vitamin E, and 15% for zinc. There was a smaller prevalence of inadequacy in other essential immune nutrients such as 11% for vitamin B6, 12% for folate, 6% for copper, 5% for iron, and <1% for selenium (Table S1).

We found that nutrient inadequacies were considerable across the major immune health vitamins, as was reported in a previous NHANES study [7]. The percentage of the population with usual intakes below the EAR in adults (≥19 years) (the 2005–2016 NHANES vs. the 2003–2007 NHANES) remained substantial for vitamin A (45% vs. 45%) and vitamin E (84% vs. 91%), with a higher prevalence for inadequacy for vitamin C (46% vs. 37%), vitamin D (95% vs. 93%), and zinc (15% vs. 11%) [7].

### 3.2. Effects of Supplement Use on Prevalence of Inadequate Intake, the 2005–2016 NHANES 

**Figure 1:**
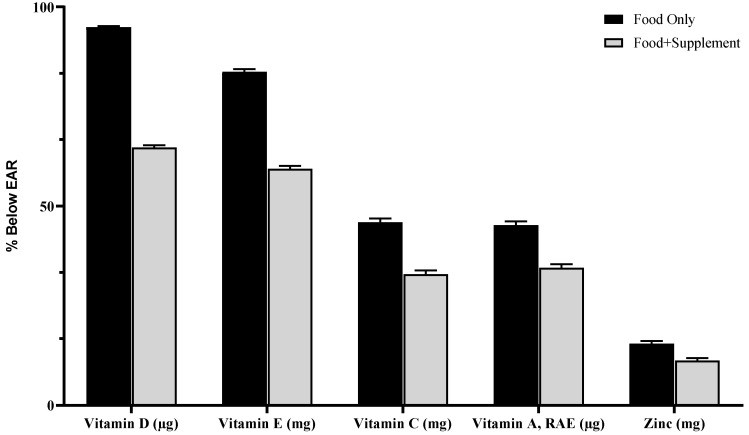
Prevalence of inadequacy (% of population below EAR) from food only and food + supplements in all adults (>19 year). The 2005–2016 NHANES.

Our research shows that food plus a dietary supplement had a lower prevalence of nutrient inadequacies than food alone. The percentage of the population with usual intakes below the EAR in adults (≥ 19 years) from food only vs. food + dietary supplements for vitamin A (45 to 35%), vitamin C (46 to 33%), vitamin D (95 to 65%), vitamin E (84 to 60%), and zinc (15 to 11%) (Figure 1).

The strength of this analysis was the use of a large, nationally representative, population-based sample (based on six NHANES cycles, 2005–2016). The limitation of the analysis was probable bias measurement errors due to self-reported data.

## 4. Role of Immune-Related Shortfall Nutrients

### 4.1. Vitamin D

For more than a century, both sunlight and dietary sources of vitamin D (cod liver oil) have been used to treat tuberculosis, a bacterial infection of the lungs [44]. Vitamin D regulates the innate as well as the adaptive immune systems. Vitamin D impacts the innate immune system in several ways. It enhances barrier function of epithelial cells in the eyes and intestinal tract [45,46]. Additionally, vitamin D enhances chemotactic, phagocytic, and bactericidal activities of key innate immune cells including monocytes, macrophages, and neutrophils [28]. Cathelicidin and defensin are antimicrobial proteins that protect the body by killing pathogens and are regulated by calcitriol, the active form of vitamin D [2,47,48,49]. Antimicrobial peptide expression in epithelial cells of the respiratory tract, helps protect the lungs from infection [49]. Vitamin D also impacts the adaptive immune system, which governs the body’s specific immune responses. Vitamin D helps facilitate the differentiation of naïve T cells into effector T cells including “killer” or “helper” T cells. Insufficient vitamin D levels impair the differentiation of naive T cells into effector T cells [50]. Vitamin D also regulates inflammation by altering the cytokine balance in favor of anti-inflammatory cytokines [32,48,51]. Interestingly, vitamin D increases the oxidative activity of macrophages, which has been linked to alternative-like polarization and production of anti-inflammatory cytokines [52]. In adaptive immunity, vitamin D inhibits T cell proliferation of Th1-cells and enhances proliferation of Th2 cells—the balance of which is thought to be an important regulator of autoimmunity [23]. Additionally, vitamin D has been shown to promote regulatory T cells, which actively suppress inflammation [19]. Thus, vitamin D plays roles in strengthening the body’s barriers and innate immune defenses, while also regulating inflammation to support healthy immune responses.

#### 4.1.1. Clinical Relevance

Maintaining adequate levels of vitamin D is important for optimal immune health. Low vitamin D status is a global public health problem in developed as well as developing countries, with approximately 1 billion people worldwide with deficient or insufficient levels of vitamin D [53]. Vitamin D does not occur naturally in many foods and people generally do not get enough sun exposure to produce adequate levels of vitamin D. Vitamin D status tends to be at its lowest in late winter and spring [54]. Declining levels of vitamin D may be linked to the seasonal prevalence of common viral infections. Accordingly, UVB exposure (representing vitamin D status) was negatively correlated with case fatality rates during the 1918–1919 influenza pandemic [54]. The “Spanish flu” pandemic killed more than 20 million people worldwide. Vitamin D activates the vitamin D receptor (VDR) to produce cathelicidin, which has antimicrobial activities in the lungs. An additional benefit of Vitamin D is reduced proinflammatory cytokine production, which may play a protective role during infections that cause a cytokine storm [54]. To avoid worsening of respiratory infections, Edlich et al. recommended that testing and treating vitamin D deficiency in health care providers and patients [55].

Coronaviruses are a family of viruses that can cause the common cold and upper respiratory infections, severe acute respiratory syndrome (SARS) and Middle East respiratory syndrome (MERS) [56]. Recent research suggests that vitamin D may influence the severity of infection of pathogenic coronaviruses that have emerged in the 21st century, such as severe acute respiratory syndrome-related coronavirus (SARS-CoV). The VDR is highly expressed in the lung and in vivo and animal research shows it may help prevent lung injury and pneumonia [57,58,59,60]. The SARS-CoV virus enters human cells via angiotensin-converting enzyme 2 (ACE2) [58]. In animal research, vitamin D-VDR signaling attenuated lung injury by blocking the renin–angiotensin system [59]. In an experimental interstitial pneumonitis mouse model, vitamin D3 led to supersession of lung inflammation and fibrosis markers [60]. Two reports assessed global data on persons with COVID-19 and found potential associations between low vitamin D status and increased risk of COVID-19 [61,62]. Vitamin D status has been shown to be significantly associated with severity of clinical outcomes in people with COVID-19. An analysis of 212 cases of people with COVID-19 found that those with vitamin D deficiency had a 19.61-fold higher risk of having a critical outcome compared to those with sufficient vitamin D blood levels (*p* < 0.001) [63]. It has been hypothesized that vitamin D supplementation reduces the risk of morbidity and mortality related to influenza and COVID-19. Grant et al. recommended raising 25(OH)D concentration above 40 ng/mL, which requires 5000–10,000 IU/day of vitamin D3 per day [64].

The Endocrine Society defines vitamin D deficiency as a serum level 25(OH)D of <20 ng/mL (50 nmol/L), vitamin D insufficiency at levels ranging from 21 to 29 ng/mL (52–72 nmol/L) and vitamin D sufficiency at levels > 30 ng/mL (75 nmol/L). A target level of 25(OH)D 40 ng/mL (100 nmol/L) ensures the individual’s “true” vitamin D value (ensures a 30 ng/mL due to lab variability) [65]. A large body of research has linked low vitamin D levels to respiratory disorders including influenza [23]. An analysis of NHANES demonstrated an increased prevalence of upper respiratory tract infection among individuals with deficient and insufficient status of vitamin D, when compared to those with sufficient levels [66]. The association is important given that analysis of 2001–2010 NHANES data showed that 29% of the US population is vitamin D deficient (<20 ng/mL) and an additional 41% are vitamin D insufficient (<30 ng/mL) [67]. In a prospective, observational study, it was seen that individuals with 25(OH)D levels at or above 38 ng/mL had a significant reduction in acute respiratory tract infections [68]. Furthermore, in a study looking at 25(OH)D levels in patients with community acquired pneumonia, 15% of the population with 25(OH)D levels < 12 ng/mL, indicating severe vitamin D deficiency, was associated with a higher 30-day mortality compared with patients with 25(OH)D levels > 12 ng/mL (*p* = 0.004) [30].

In two randomized controlled trials, vitamin D did not reduce the risk of upper respiratory tract infections. The VIDARIS randomized controlled trial found a monthly dose of 100,000 IUs of vitamin D3 for 18 months did not reduce the risk of upper respiratory tract infection over placebo. The average 25(OH)D level at baseline was 29 ng/mL, which is considered close to a sufficient level [69]. The vitamin D Outcomes and Interventions in Toddlers trial was conducted in children aged between 1 and 5 years [70]. The study found 2000 IU vitamin D per day did not reduce risk of respiratory tract infection over 400 IU per day. Respective mean baseline blood levels of vitamin D for the two groups were 35.9 and 36.9 ng/mL, which are sufficient blood levels of vitamin D. Participants in these trials had adequate vitamin D levels, so the applicability of these trials to broader US populations is unclear given the high prevalence of vitamin D insufficiency and deficiency. A broader approach using systematic review and meta-analysis has revealed potential benefits of vitamin D supplementation. Individual participant data from 25 randomized controlled trials involving 11,321 participants age 0 to 95 year examined the effects of vitamin D on acute respiratory tract infections [71]. They found that vitamin D supplementation significantly lowered risk for acute respiratory tract infections by 12%, however, there was a 70% lower risk of respiratory infection with vitamin D supplementation in participants whose baseline 25(OH)D levels were <10 ng/mL than in those with baseline 25(OH)D levels >10 ng/mL.

#### 4.1.2. Current Findings

A large body of research shows a growing concern of the impact of inadequate intake of vitamin D and suboptimal/deficiency status on immune health. Our findings contribute to this concern of vitamin D inadequacy. Our data show that the percent of the population with intake below the EAR of 400 IU (10 μg) was 95% (food only) and 65% (food + supplement). Vitamin D usual intake was 188 IU (4.7 µg) (food only) and 556 IU (13.9 µg) (food + supplement) (Table S1). The RDA for vitamin D is 600–800 IU (15–20 μg)/day [72]. However, this recommendation is limited to bone health. The Endocrine Society recommends 1500–2000 IU (38–50 μg) to maintain a minimum of serum 25(OH)D concentration of 30 ng/mL [65]. For adults aged 19 years and older, higher doses (up to 10,000 IU/day), may be necessary to correct, treat, and prevent vitamin D deficiency [65].

### 4.2. Vitamin E

Vitamin E is a potent lipid-soluble antioxidant that influences immune health. Its importance in immune function is evident by the fact its concentration is up to 30-fold higher in white blood cells than in red blood cells [73]. Vitamin E’s main role in every step of the immune system is as a lipid soluble antioxidant that protects the cell membranes against lipid peroxidation from free radical attack. In the innate immune system, vitamin E supports epithelial barrier by protecting cell membranes from oxidative damage. It has been shown to improve the function of many innate immune cells, such as cytotoxic activity of natural killer (NK) cells, as well as neutrophil movement and phagocytosis [74]. Phagocytes produce reactive oxygen species (ROS) to help kill pathogens and vitamin E helps quench these oxidative bursts to prevent cellular damage. One study showed that vitamin E supplements increased phagocytic capacity, but decreased bactericidal activity, which may due to vitamin E’s function as an antioxidant. [75,76]. Vitamin E influences dendritic cell activity, which links innate and adaptive immune systems [74]. In the adaptive immune system, vitamin E enhances lymphocyte proliferation, interleukin-2 (IL-2) production, and inhibits production of prostaglandin E2 (PGE2), which indirectly protects the function of T cells [2]. Vitamin E improves efficacy in immune synapse formation in naive T cells to ensure activation of T cell signals and modulates Th1/Th2 balance [74].

#### 4.2.1. Clinical Relevance

One of vitamin E’s most studied effects in immunity is prevention of immunosenescence seen with aging. In aging, there is a decline in T cell-mediated immunity caused by thymic involution, decreased naive T cells and decreased IL-2-simulated T cell proliferation capacity [39]. Vitamin E deficiency impairs immune function in both animal and human studies and the repletion of vitamin E is able to restore it [39].

Meydani et al. showed that giving 800 IU vitamin E/day for 1 month to healthy adults over 60 years of age had reduced levels of lipid peroxides and improved measures of immune function including, increased delayed-type hypersensitivity response and ex vivo T cell proliferation [33]. A follow-up trial showed that vitamin E (200 IU/day of dl-α-tocopherol) improved efficacy of hepatitis B and tetanus vaccines in older adults [77]. These results were confirmed in another study that showed 200 IU/day vitamin E also improved markers of both innate and adaptive immune function [18].

In several human clinical studies, Vitamin E supplementation has shown a clinical benefit in reducing respiratory infections in older adults. In older adults with pneumonia, those who received vitamin E supplements had an associated lower rate of self-reported rehospitalization [78]. In a randomized, double-blind, placebo-controlled trial, 600 nursing home residents (≥65 years old) who supplemented with 200 IU vitamin E/day (dl-α-tocopherol) vs. placebo reduced the incidence of upper respiratory infection by 20% [34]. In elderly adults, vitamin E supplementation did not impact self-reported respiratory infections, although participants with absence of respiratory infection were not confirmed, which may have resulted in reporting biases [22].

Although most clinical research on vitamin E shows an effective reduction in risk of viral infections of the upper respiratory tract (mostly the common cold), it may hold promise for a protective role against influenza viruses. Influenza viruses induce significant oxidative burst in the lungs, which can rapidly result in organ damage and impaired function [79]. Vitamin E is the most prevalent scavenger of lipid peroxyl radicals in the lipid membrane and quickly breaks peroxyl chain propagation reactions in vivo [79]. In animal studies, vitamin E was the most efficient antioxidant at reducing lipid peroxidation levels induced by influenza virus A infection [79]. More research is needed to understand the role of vitamin E in influenza virus infections.

It is estimated that ~34% of the US population has metabolic syndrome [80] and recent studies indicate vitamin E needs may be higher in people with metabolic syndrome [81]. Evidence also suggests that individual components of metabolic syndrome, such as obesity and diabetes are associated with higher mortality related to H1N1 influenza [82,83]. Recent data has suggested that diabetes, hypertension and cardiovascular disease worsens COVID-19 infections [84]. Since individuals with metabolic syndrome may be more vulnerable to infection-related severity and/or mortality, future research is needed to understand whether optimizing vitamin E intake levels can improve immune response, as seen in older populations.

#### 4.2.2. Current Findings

Our data show that the percent of vitamin E intake below the EAR (18 IU/12 mg) was 84% (food only) and 60% (food + supplement). The prevalence of inadequacy of 84% has decreased from previous NHANES data that showed a 91% inadequacy [7]. Vitamin E usual intake was 9 mg (food only) and 28 mg (food + supplement) (Table S1). The RDA for vitamin E is 15 mg/day (22.4 IU) [85] and optimal daily intake for immune health for older adults is 134 mg/day (200 IU) [86]. Additionally, there is an emerging area of research around higher vitamin E needs in people with metabolic syndrome, who are at greater risk of infection [81].

### 4.3. Vitamin A

Vitamin A plays a role in innate and adaptive immunity. From the early days of its recognition in the early 20th century, vitamin A was associated with resistance to infection [87], to the extent that it was referred to as “the anti-infective vitamin”. In innate immunity, it is responsible for helping maintain the physical barriers of the body—the skin, and epithelial mucosal cells of the respiratory and GI tracts. Vitamin A is essential for directing innate immune cells to the mucosa in the intestine, where they reside to help prevent the entry of pathogens [88]. Vitamin A plays vital roles in the regulation of the various aspects of innate immune cells, such as differentiation, maturation and function [89]. It regulates the function of NK cells and macrophage phagocytosis [2]. Recent reports show that retinoic acid plays a role in dendritic cell differentiation, the key antigen-presenting cells for activating naive T cells [90]. In adaptive immunity, vitamin A is involved in the development and differentiation of Th1 and Th2 cells and the normal function of B cells [2].

#### 4.3.1. Clinical Relevance

Several human studies have linked low plasma or serum vitamin A to impairment in immunity [89]. Most clinical studies on vitamin A have been limited to children with vitamin A deficiencies. Vitamin A deficiency is a global public health problem and increases risk of infectious diseases [89]. Vitamin A supplementation on a large scale in third-world countries is a cost-effective intervention to improve vitamin A status [91]. In a meta-analysis that included 43 randomized controlled trials studying children 6 months to 5 years of age found a 24% reduction in risk of all-cause mortality due to vitamin A supplementation. Measles morbidity was reduced by 50%. However, there was no impact on incidence of respiratory disease or hospitalizations due to diarrhea or pneumonia [92]. In HIV-infected adults, low vitamin A status has been associated with increases in disease progression and mortality, however results from studies are inconsistent [93].

#### 4.3.2. Current Findings

Our data show that the percent of the population with intakes below the EAR (500–625 μg) for vitamin A was 45% (food only) and 35% (food + supplement). Vitamin A usual intake was 639 μg (food only) and 977 μg (food + supplement) (Table S1). The RDA for vitamin A is 700–900 μg/day [85].

### 4.4. Vitamin C

Vitamin C contributes to both the innate and adaptive immune system. In the first line of innate immunity, vitamin C supports the epithelial barrier though synthesis of collagen and protection from ROS [12]. Vitamin C stimulates production and function of leukocytes, enhances chemotaxis and clears left-over debris from neutrophil attack (neutrophil extracellular trap) [12]. Throughout the innate system, vitamin C is an effective water-soluble antioxidant that protects cells by combating ROS generated by immune cells and regenerates oxidized glutathione and vitamin E. In adaptive immunity, vitamin C plays a role in B and T lymphocyte differentiation and proliferation, possibly through gene regulation. It increases circulating immune system defenses including antibodies and complement proteins [12].

#### 4.4.1. Clinical Relevance

Extensive research has shown that vitamin C is a pivotal nutrient in immune health. Almost 150 animal studies have shown that vitamin C may alleviate or prevent bacterial or viral infections [94]. Numerous clinical trials have investigated Vitamin C supplementation for the prevention and treatment of the common cold with conflicting findings due to various study designs and differing doses. Very few studies on vitamin C and the common cold have measured vitamin C status. A study conducted on 28 healthy men between 18 and 35 years with plasma vitamin C < 45 µmol/L showed that 1000 mg vitamin C per day taken for eight weeks reduced duration of colds by 59% compared to placebo [95]. In 2013, a Cochrane review that included 24 trials found that vitamin C (at least 200 mg) did not significantly reduce the risk of common cold in the general population, but found a 52% reduction in cold in 5 trials with 598 marathon runners, skiers and soldiers engaged in subarctic exercises, indicating a higher need for those exposed to physical stress. In the general population, although there was no risk reduction in the common cold, at least 200 mg vitamin C reduced cold duration by 8% in adults and 14% in children. In children, 1 to 2 g/day vitamin C shortened colds by 18%. When taken after the onset of cold symptoms, vitamin C (1–8 g) did not affect cold duration or symptom severity [96].

Vitamin C may help prevent age-induced impairments of immune function. Preclinical researchers found high vitamin C intake in mice inhibited the age-related thymic involution and maintained T cell production, which is key to preventing the decline in T cell function commonly seen with age [36]. Low vitamin C status has been observed in the independent and institutionalized elderly population indicated by lowered plasma and leukocyte vitamin C concentrations. Low vitamin C plasma level concentrations (<17 µmol/L) in people aged 75–84 years was a predictor of all-cause mortality [97]. Research has shown vitamin C (500 mg–1 g) can improve vitamin C status and generation of ROS by neutrophils [27]. Clinical trials found vitamin C beneficial in patients with pneumonia with low vitamin C status. A randomized double-blind trial showed that vitamin C (200 mg) taken for 4 weeks in 57 elderly hospitalized pneumonia patients reduced the respiratory symptom score in those with low vitamin C levels [98]. Previous research in pneumonia patients showed that vitamin C (250 mg–800 mg/day) reduced days in hospital by 19%, whereas higher dosing of vitamin C (500 mg–1.6 g/day) reduced days in hospital by 36% compared to a placebo [94].

#### 4.4.2. Current Findings

Our findings show that the percent of intake below the EAR (60–70 mg) for vitamin C was 46% (food only) and 33% (food + supplement). The prevalence of inadequacy of 46% is higher than previous NHANES data that showed a 37% inadequacy [7]. Vitamin C usual intake was 83 mg (food only) and 168 mg (food + supplement) (Table S1). The RDA for vitamin C is 75–90 mg/day [85]. Optimal daily intake is at least 200 mg/day, the amount to reach 60 μmol/L for optimal cell and tissue levels [99] and minimum dose found to decrease the duration of the common cold [96].

### 4.5. Zinc

Normal development and function of the innate and adaptive immune system relies on zinc [100]. In innate immunity, zinc helps maintain mucosal integrity. It is a cofactor for many metalloenzymes that are involved in maintenance of the cell membrane [2]. Zinc enhances the phagocytic activity of NK cells, monocytes, neutrophils and macrophages [29]. In the adaptive immune system, zinc is a major player in T cell development, differentiation, and T cells and B cells activation signaling. It induces proliferation of T helper (Th1) and T killer cells which help coordinate and attack pathogens. Zinc also induces development of regulator T cells (Tregs) which help control the immune response by suppressing T cell proliferation and pro-inflammatory cytokine production. Collectively, this helps maintain homeostasis and self-tolerance, which is a critical role in preventing autoimmunity [37]. Zinc acts as a co-factor for enzymes that protect against oxidative damage as part of the antioxidant defense system [101].

#### 4.5.1. Clinical Relevance

The critical need for adequate zinc intake for maintaining the integrity of the immune system has been shown in animal and human studies. The impact of inadequate zinc intake on the immune system is rapid and extensive. Mouse models demonstrate that low zinc intake can lead to impaired immune function [20]. Zinc deficiency causes thymic involution, leading to reduced T cell production and an imbalance of T helper cells (Th2 > Th1) and an altered cytokine production which can lead to increased inflammation and oxidative stress. These changes mimic the changes seen in the aging immune system, so even marginal zinc intake in older adults may have a deleterious impact on immune health [24]. An earlier analysis found that 35–45% of US adults > 60 years had zinc intakes below the EAR [102]. Previous reports that plasma zinc concentration declines with age may be due to decreased absorption or uptake, or epigenetic alterations [24,38].

Several randomized controlled trials and meta-analyses have shown that 10–45 mg zinc/day improves several aspects of immune function in older adults. A randomized, double-blind, placebo-controlled trial showed 45 mg zinc (zinc gluconate) in forty people aged 56–83 years reduced IL-6, secretory phospholipase A, malondialdehyde and hydroxyalkyls, markers of inflammation and oxidative stress [103]. Another randomized, double-blind, placebo-controlled trial in 100 older adults (aged 50–70 years) with normal blood zinc concentrations gave 15–30 mg zinc supplementation for six months. Lower doses of zinc (15 mg zinc/day) significantly increased the proportion of helper T cells to that of cytotoxic T cells at six months [25]. Maintaining adequate zinc status may help prevent immune dysfunction in the older nursing home population. A one-year study of 420 nursing home patients found that participants with plasma concentrations of zinc >70 μg/dL had a lower risk of pneumonia [104]. Several clinical studies have shown zinc supplementation may reduce risk of common cold, although results are mixed. A recent meta-analysis of seven randomized trials with 575 participants with naturally acquired common colds concluded that zinc (zinc gluconate or zinc acetate, 80 to 92 mg per day) supplementation starting at the beginning of the first cold symptoms resulted in a 33% reduction in cold duration. Zinc acetate lozenges shortened colds by 40% and zinc gluconate by 28%. Zinc supplementation at 192–207 mg/day had a similar effect on cold duration [105]. Researchers are beginning to explore the effect of zinc on influenza virus. A recent in vitro study showed that zinc oxide had inhibitory effects on H1N1 influenza virus [106]. Future research is needed to extend these finding to in vivo models.

#### 4.5.2. Current Findings

Our research shows, for all adults (≥19 years), the percent of intake below the EAR (6.8–9.4 mg) for zinc was 15% (food only) and 11% (food + supplement). The prevalence of inadequacy of 15% is slightly higher than previous NHANES data, which showed a 11% inadequacy [7]. Zinc usual intake was 12 mg (food only) and 16 mg (food + supplement) (Table S1). The RDA for vitamin zinc is 8–11 mg, which is adequate for healthy populations [107]. The optimal daily intake for immune health for older adults at high risk for infection has been suggested at 30 mg/day [16].

## 5. Food and Dietary Supplements to fill Nutrient Gaps

Ideally, a healthy, nutrient-dense diet, including a variety of colorful fruits and vegetables (vitamin A and C), whole grains, nuts, seeds, oils (vitamin E), meats and legumes (zinc), and dairy and seafood (vitamin D) can provide the nutrients needed to meet daily requirements (Table 1). However, research shows Americans are not eating the foods necessary to meet their needs for key micronutrients [108] which has contributed to micronutrient inadequacies that have been reported for almost 15 years [6,7,109]. Findings from our data show a decade of substantial shortfalls in nutrients that support immune health (vitamin A, C, D, E) and that some shortfalls are higher (vitamin C, D, and zinc) than previously reported [7].

Studies looking at the effects of dietary supplements on immune health have been inconsistent, which may be due to failure to control for factors such as measuring baseline nutrient status, which eliminates groups with inadequacy who can benefit the most from the nutrient. Other factors that may impact outcome are timing and duration of the nutrient dose. This was seen in the vitamin C Cochrane review. Vitamin C was only effective as a continuous prophylaxis, but had no impact if taken after the onset of cold symptoms [96].

Our research shows that food plus a dietary supplement had a lower prevalence of nutrient inadequacies than food alone. Previous research has shown food fortification [7,110] and the consumption of dietary supplements increase overall nutrient intake and decrease the prevalence of nutrient inadequacies [6,7,109]. Specifically, Blumberg et al. showed that the use of a multivitamin/mineral supplement (at least 100% of the RDA or AI for at least nine nutrients), compared with food alone, was associated with a lower prevalence of inadequacy for most 15 nutrients (including vitamins A, C, E and zinc), except for vitamin D [6]. Those with the highest compliance (>21 days per month) had the most impact on eliminating inadequacies and lowered the odds ratios of deficiency for most blood biomarkers.

A multivitamin/mineral supplement is an excellent tool, as an addition to a healthy diet, to ensure adequate intake of essential nutrients for general health. A multivitamin/mineral supplement that offers 100% of the RDA can fill the nutrient gaps of most essential nutrients for immune health but may fall short of optimal levels recommended by scientific groups for vitamin C (200 mg) [99] and vitamin D (1500–2000 IU) [65]. Older adults or those with suboptimal status may have higher needs in vitamin E and zinc, as well. For those with a vitamin D insufficiency/deficiency, higher doses may be required to raise vitamin D levels to a minimum of serum 25(OH)D concentration of 30 ng/mL [65]. In such cases, higher dose dietary supplements may be needed to reach optimal levels.

## 6. Conclusions

A well-functioning immune system is essential to survival. Micronutrients are a fundamental part of the immune system and require optimal levels for effective immune function. Nutrient insufficiency/deficiency of one nutrient can adversely affect immune health, while multiple inadequacies may put the immune system at a bigger deficit. The prevalence of inadequate nutrient intakes that support the immune system (vitamin A, C, D, E) remain substantial and some are higher (vitamins C, D, and zinc) than previously reported [7]. Given the long-term and widening of nutrient gaps in the U.S.—specifically in critical nutrients that support immune health—public health measures should adopt guidelines to ensure an adequate intake of these micronutrients, especially in vulnerable populations. Future research is needed to better understand the interactions and complexities of multiple nutrient shortfalls on immune health and assess and identify optimal levels of intake and nutrient status in at-risk populations. The objective of such a platform will not only decrease the prevalence of inadequacy but move towards a focus on optimal health and well-being.

## Figures and Tables

**Table 1:** Nutrient Daily Recommendations and Food Sources.

Nutrient	EAR	RDA	Optimal Intake	Food Sources [111]
**Vitamin A**				
	500–625	700–900	N/A	Beef liver, sweet potato, spinach, pumpkin and carrots
	μg RAE	μg RAE		
**Vitamin C**				
	60–70 mg	75–90 mg	200 mg [99]	Red pepper, orange juice, kiwifruit, broccoli, strawberries
**Vitamin D**				
	400 IU(10 μg)	600–800 IU (15–20 μg) Skeletal health	1500–2000 IU (38–50 μg) [65] Dose to maintain a blood level of 25(OH)D ≥ 30 ng/mL	Trout, salmon, mushrooms, milk (vitamin D fortified), Ready-to-eat cereal, fortified with at least 10% of DV for vitamin D, eggs
**Vitamin E**				
	12 mg(18 IU)	15 mg (22.4 IU)	Older adults: 134 mg (200 IU) [86]	Sunflower seeds, almonds, safflower oil, peanut butter, spinach
**Zinc**				
	6.8–9.4 mg	8–11 mg	Older adults:	Oysters, crab, beef, baked beans, yogurt
			30 mg [16]	

## Supplementary Materials

The following are available online at https://www.mdpi.com/2072-6643/12/6/1735/s1, Table S1: Micronutrient usual Intake (UI), prevalence of inadequacy (% of population below EAR) and % of population exceeding the UL from food only and food + supplements in all adults (>19 year). The 2005–2016 NHANES.
