# Supplementary material for: Inadequacy of Immune Health Nutrients: Intakes in US Adults, the 2005–2016 NHANES
Source: Nutrients. 2020 Jun 10;12(6):1735. doi: 10.3390/nu12061735 (PMC7352522; doi:10.3390/nu12061735)
Supplement: Supplementary file 1 [file nutrients-12-01735-s001.pdf]

Table S1. Micronutrient usual Intake (UI), prevalence of inadequacy (% of population below EAR) and % of population exceeding the UL from Food Only and Food + Supplements in all adults (> 19 y). NHANES 2005 – 2016.

Nutrients	Usual Intake		% of Population Below EAR		% Population Above UL	
	**Food Only** n = 26,282	**Food+Supplement** n = 21,248	**Food Only** n = 26,282	**Food+Supplement** n = 21,248	**Food Only** n = 26,282	**Food+Supplement** n = 21,248
Calcium (mg)	973.44 ± 6.01	1141.55 ± 9.02	42.91 ± 0.68	31.20 ± 0.71	0.54 ± 0.05	4.27 ± 0.21
Copper (mg)	1.30 ± 0.01	1.64 ± 0.01	6.05 ± 0.36	4.60 ± 0.34	<0.01	0.03 ± 0.01
Iron (mg)	15.01 ± 0.08	18.42 ± 0.14	5.20 ± 0.22	3.98 ± 0.21	0.01 ± 0.00	1.79 ± 0.10
Magnesium (mg)	305.43 ± 1.98	335.50 ± 3.11	53.46 ± 0.85	45.61 ± 0.96	-	-
Phosphorus (mg)	1390.39 ± 6.46	1402.48 ± 6.84	0.80 ± 0.11	0.76 ± 0.12	0.01 ± 0.00	0.01 ± 0.00
Selenium (mcg)	114.33 ± 0.61	129.55 ± 0.76	0.50 ± 0.09	0.40 ± 0.08	<0.01	0.12 ± 0.02
Zinc (mg)	11.61 ± 0.07	15.93 ± 0.15	15.41 ± 0.71	11.21 ± 0.63	<0.01	2.21 ± 0.14
Vitamin A (mcg)[1]	639.36 ± 7.45	977.43 ± 15.44	45.21 ± 0.96	34.52 ± 0.89	<0.01	2.09 ± 0.17
Thiamin (mg)	1.63 ± 0.01	5.76 ± 0.40	6.57 ± 0.46	4.61 ± 0.38	-	-
Riboflavin (mg)	2.18 ± 0.01	4.69 ± 0.18	2.86 ± 0.18	2.24 ± 0.17	-	-
Niacin (mg)	26.00 ± 0.14	36.61 ± 0.74	1.43 ± 0.16	0.91 ± 0.13	16.29 ± 0.45	34.63 ± 0.60
Vitamin B6 (mg)	2.12 ± 0.01	5.40 ± 0.17	11.07 ± 0.56	7.44 ± 0.48	<0.01	0.76 ± 0.09
Folate DFE (mcg)	541.33 ± 3.92	763.51 ± 7.51	12.05 ± 0.58	8.18 ± 0.51	-	-
Folic acid (mcg)	188.41 ± 1.70	318.55 ± 3.65	-	-	<0.01	1.86 ± 0.14
Vitamin B12 (mcg)	5.20 ± 0.05	56.79 ± 3.17	3.95 ± 0.31	2.57 ± 0.22	30.44 ± 0.77	53.07 ± 0.69
Vitamin C (mg)	82.88 ± 1.18	168.53 ± 4.06	45.92 ± 0.94	32.90 ± 0.92	<0.01	0.46 ± 0.09
Vitamin D (mcg)[2]	4.71 ± 0.05	13.88 ± 0.37	94.90 ± 0.31	64.69 ± 0.56	<0.01	1.34 ± 0.15
Vitamin E (mg)[3]	8.64 ± 0.08	27.76 ± 1.00	83.70 ± 0.67	59.34 ± 0.77	-	-
**Nutrients with AI, (% above)**						
Vitamin K (mcg)	110.63 ± 1.73	116.88 ± 2.35	44.92 ± 1.17	48.51 ± 1.34	-	-
Total choline (mg)	335.73 ± 1.69	336.51 ± 1.91	7.55 ± 0.47	7.70 ± 0.50	<0.01	<0.01

1 Vitamin A as mcg RAE (retinoic acid equivalents); 2 Vitamin D as mcg of Vitamin D2+Vitamin D3; 3 Vitamin E as mg of αtocopherol.
